# On the Origin of Sequence

**DOI:** 10.3390/life5041629

**Published:** 2015-11-16

**Authors:** Peter T. S. van der Gulik

**Affiliations:** Centrum Wiskunde & Informatica, P.O.Box 94079, 1090 GB Amsterdam, The Netherlands; Peter.van.der.Gulik@cwi.nl; Tel.: +3120-5924232; Fax: +3120-2031226

**Keywords:** origin of life, peptides, prebiotic processes, error robustness, genetic code

## Abstract

Three aspects which make planet Earth special, and which must be taken in consideration with respect to the emergence of peptides, are the mineralogical composition, the Moon which is in the same size class, and the triple environment consisting of ocean, atmosphere, and continent. GlyGly is a remarkable peptide because it stimulates peptide bond formation in the Salt-Induced Peptide Formation reaction. The role glycine and aspartic acid play in the active site of RNA polymerase is remarkable too. GlyGly might have been the original product of coded peptide synthesis because of its importance in stimulating the production of oligopeptides with a high aspartic acid content, which protected small RNA molecules by binding Mg^2+^ ions. The feedback loop, which is closed by having RNA molecules producing GlyGly, is proposed as the essential element fundamental to life. Having this system running, longer sequences could evolve, gradually solving the problem of error catastrophe. The basic structure of the standard genetic code (8 fourfold degenerate codon boxes and 8 split codon boxes) is an example of the way information concerning the emergence of life is frozen in the biological constitution of organisms: the structure of the code contains historical information.

## 1. Introduction

In this review we consider four interesting topics in succession. The first of these is the improbability of the origin of life. The second is the origin of the feedback loop underlying life. This is followed by error robustness, the third topic. The fourth and last topic of this review is the information frozen in the genetic code. Of course, the four topics are closely related.

## 2. The Improbability of the Origin of Life

The subject of the theme issue of which this review is a part is the emergence of life, a subject of particular interest to the general public and also an important issue with regard to present-day space exploration (see below). This theme issue is split into several sections, such as origin of building blocks, emergence of sequences, and relationship between proteins and nucleic acids; but, according to the line of thought presented in this review, these aspects of the emergence of life are all closely related. However, before we consider these relations, we first have to focus on the improbability of the origin of life. With the astounding development of the technical and mathematical legacy of Galileo, both in optics and in mechanics, we now are able to discover many planets in systems outside our own Solar System. Special attention is paid to the planets present in the habitable zone of their system, where it is not too hot, and not too cold, and water is present in fluid form. In fact, the study of space sometimes looks like a search for extraterrestrial life, at present. It is important to realize that finding a planet with fluid water is not the same as finding a planet with life. Our planet Earth is a very special planet, and that is not only because it is not too hot, not too cold, and not too dry. The attitude of expecting to find life after having found water resembles the attitude of expecting to find life after having found amino acids. Stumbling on the constituents is not the same as stumbling on life! To make a cake, more is necessary than just the ingredients. One needs also a recipe, and an oven. In the same way, finding hydrogen does not mean that one has found life. This also goes for finding water and for finding amino acids.

**The mineralogical composition**. There are three aspects of planet Earth which are very special. The first of these three aspects is the mineralogical composition. Earlier this year, Hystad *et al.* [1] convincingly argued that the mineralogical composition of Earth is not just rare in the Universe, but is unique in the Universe. Although the existence of a large part of the rare minerals on Earth is caused by the presence of life [1], the existence of another part of the rare minerals on Earth is caused by physical/chemical factors alone, and it is possible that some of these rare minerals were a causal factor in the emergence of life. Hystad *et al.* discuss the special case of Be containing minerals; from the perspective of biology, B is a more interesting element than Be to take into consideration [2]. B containing compounds are able to prevent sugar reactions to go beyond 5-carbon sugars such as ribose [2,3,4]. However, B is just an example. Rare minerals containing elements other than B may have played a decisive role in the emergence of life. Until the role of these minerals in that process is better understood, it is not realistic to look for life when all one has found is water.

**The Moon**. The second aspect of planet Earth that is very special is the Moon. In fact, “planet Earth” is a scientifically unacceptable simplification. In our Solar System, we have many subsystems, and one of these subsystems is the Earth-Moon-Double-Planet-System. The Moon is, when compared to satellites of other planets in our Solar System, special in being in the same size category as Earth. This means that the Moon has a considerable gravitational influence on the Earth. The subsystem emerged as a consequence of a special event early in the history of the Solar System (see e.g. [5]). One of the consequences of the gravitational influence of the Moon, which was present in the era in which we (generally) envision the emergence of life, is the presence of tidal cycles. When considering the origin of life, a realistic possibility is that ebb/flow cycles caused by the Earth-Moon interaction produced PCR-like (Polymerase Chain Reaction-like) processes, which possibly played an essential role in the origin of life. Another effect of tidal cycles is hydration/dehydration cycles (see e.g. [6] and references therein). Unless it is ruled out that tidal circumstances were essential for the emergence of life, it is not realistic to look for life when all one has found is water.

**The triple environment**. The third aspect of our Earth-Moon-Double-Planet-System that is very special is the relative abundance of the three environments of ocean, atmosphere, and continent on Earth. None of the three is present in over-abundance compared to the other two, and this means that (1) there are a lot of interface situations (our own human existence is largely at one of these: the continent/atmosphere interface); (2) products which have been formed in one of these environments can diffuse into one of the other two, and encounter radically different circumstances there (see e.g. [7]). It is a realistic possibility that this special aspect was essential for the emergence of life (for an example of the possible importance of interfaces, see [6]). Taking these three special aspects (the possibly unique mineral composition, the Moon, and the triple environment) together, one can conclude that it possibly is wishful thinking to strive to find extraterrestrial life in time and space close enough to the Solar System to be detectable. These considerations should be kept in mind when studying the role of small peptides in the emergence of life: the circumstances in which life emerged possibly were rare in the Universe.

## 3. The Origin of the Feedback Loop

It is amazing that the enzymatic activity of the dipeptide GlyGly is not generally known among researchers interested in the origin of life. Glycine is the smallest of the Set of Twenty, and the fact that the smallest polymer of the simplest amino acid has an enzymatic function, and a very relevant one, should not stay unnoticed. GlyGly has amino acid polymerizing activity [8,9,10], and thus can, in a certain way, be considered as the most primitive ribosome. Here we see a very small sequence involved in very primitive “protein synthesis”. Large and complex things often started as small and simple things, and gradually grew in size and complexity. Taking into consideration how important the activity of GlyGly is, coded protein synthesis might have originated as coded production of GlyGly.

If uncoded oligopeptide synthesis via the Salt-Induced Peptide Formation (SIPF) process [11] and enhanced by GlyGly [8] was so important that it led to the coded synthesis of GlyGly, what could have been the function of these uncoded oligopeptides? A possible answer is: binding Mg^2+^ ions. One of the most important active sites in biology is the one which produces mRNA. This active site is AsnAlaAspPheAspGlyAsp in all organisms, without exception. The three C-terminal residues in this active site are the most important. The two aspartic acid residues are involved in binding the catalytic Mg^2+^ ion, and the glycine residue is able to let the string make the precise turn to be able to do that, all other members of the Set of Twenty are too big to fulfill that position. Aspartic acid and glycine were (together with alanine) the proteinogenic amino acids present in larger amounts in Miller’s very first experiment. Here we encounter a second fact which is (amazingly!) not generally known among researchers interested in the origin of life: the very heart of mRNA production consists of amino acids which are as primordial as glycine and aspartic acid [12]. Of course, aspartic acid is, in Miller-Urey experiments, always much less abundant than glycine. Here it is important to take into consideration that certain clay species (like montmorillonite) enhance the SIPF reaction [13]. The positively charged surface of such a clay will enrich the aspartic acid content in the reactions compared to the glycine content because of the two carboxylic acid groups of aspartic acid compared to just one of glycine. The uncoded oligopeptides which were that important (that they led to the coded synthesis of GlyGly), were oligopeptides containing glycine and aspartic acid, able to bind Mg^2+^ ions, and ultimately leading to the synthesis of RNA oligomers, this is the hypothesis proposed in [14].

The RNA oligomers were proposed to be able to produce, in a coded way, GlyGly (for further details concerning binding, recognizing, and coding the reader is referred to [14]; the present review focuses on the feedback loop itself). By doing this, they protected their own existence (see for the protection concept [15]) and, in a slightly further evolved stage, promoted their own production [14]. This feedback loop (GlyGly produces AspGlyAsp and AspGlyAsp-like sequences; these in turn protect and produce RNA; RNA produces GlyGly) is the secret of life. This is the essence, this is where it starts, and this is the sine qua non. This enables larger, macromolecular sequences to emerge. Likely the RNA sequences were the first that grew larger and that explored the possibilities of new kinds of catalysis; in this respect we should keep Schultes’ and Bartel’s discovery in mind [16] that in RNA sequence space, different catalytic activities are found in rather close proximity. The oligopeptide component of this coevolving living system at first likely remained smaller in molecular size; the function of oligopeptides was, however, essential. This should be kept in mind when we consider the “RNA world” [14]. After RNA catalysis had made the system sophisticated enough to maintain larger genetic memory, coded peptide sequences could start to grow. The oligopeptides were able to form isolated active sites, when chemically cooperating with metal ions [12]. This is true for the “enzymatic” function of GlyGly (see e.g. [8]) as well as for that of the different aspartic acid containing oligopeptides. The power of such isolated active sites was fundamental to life, but, seen from an advanced biochemical perspective, rather restricted. When peptide sequences arrived in the era of the gigantic (speaking from molecular perspective) macromolecules, proteins were able to form the structures consisting of alpha-helices and beta-sheets, and with help of these macromolecular skeletons establish superb molecular recognition power. Ribozymes were then soon driven into obscurity. However, the original function of coded synthesis was not providing macromolecular enzymes but providing oligopeptide catalysts.

**Storage compounds**. One should not forget the possible further functions of coded oligopeptides. The second step in evolving the genetic code (after just rough production of GlyGly) proposed in this study, is production of AlaAla. This was not because AlaAla could do something remarkable, like GlyGly. This was because alanine was present in large amounts, and too often alanine was incorporated in the dipeptides instead of glycine. By making specific AlaAla molecules, the quality of GlyGly production was improved, because some alanine “pollution” was taken out of the GlyGly production. Those effects were the ones that counted, in early biology. Now, we have seen the emergence of a spandrel, in Stephen Jay Gould’s beautiful words. Something has emerged, and next evolution finds a function for it. This is called exaptation. The proposal here is that AlaAla became the first storage compound. The nice thing about storage is that the exact sequence is not that important. AlaAlaGlyAla is a good storage oligomer, and GlyAlaAlaAla too, and also AlaAlaAlaGly. Later, alanine became a good linker. GlyAlaAlaAlaGly could be an example of a molecule that can bind nucleic acid with the backbone nitrogen atoms of the glycine residues. The alanine residues in the middle provide the space between the different binding sites. An amino acid repertoire consisting of just glycine and alanine has been proposed before (see [17] and references therein).

**Metabolism**. These oligopeptides likely evolved in coevolution with metabolism. Some parts of central metabolism might be ancient. Glycine is connected to alanine via part of the glycolysis, via a graph consisting of the nodes serine, phosphoserine, 3-phosphoglycerate, 2-phosphoglycerate, phosphoenolpyruvate and pyruvate. Alanine is also connected to aspartic acid via part of the glycolysis and part of the TCA cycle, another graph. These reactions connecting glycine, alanine, and aspartic acid might be the oldest parts of metabolism. Using these metabolic connections, the abundance of alanine could be funneled into glycine presence and aspartic acid presence. A very important thing to keep in mind is that the central part of the purine double ring is derived (in biosynthesis) from glycine, and the largest part of the pyrimidine ring is derived (in biosynthesis) from aspartic acid. These biosynthetic steps are universal, and it is therefore likely that these steps were evolved by the living world when growth depleted prebiotic building blocks of RNA. In the early RNA world, amino acids were thus playing roles in biology other than their role in polypeptides alone (and it is good to remember amino acids are still playing many of these early roles: amino acids are more than just protein constituents). Alanine atoms ending up in RNA (via conversion of alanine in glycine or aspartic acid) of course means better (less “polluted”) GlyGly production.

The concept that oligopeptides evolved in coevolution with metabolism was already introduced in evolutionary biochemistry in 1975, as the coevolution theory of the origin of the genetic code [18] (see also [19] and references therein). In this theory, early peptides consisting of a limited repertoire of amino acids (like glycine, alanine, and aspartic acid) were able to expand the metabolic pathways and develop synthetic routes for more complex amino acids (like proline, leucine, and glutamic acid). With this expanded repertoire of amino acids, a more sophisticated biochemistry could develop, and even more complex amino acids (like histidine, tyrosine, and tryptophan) could become part of biochemistry. The present-day highly complex biochemistry thus developed in a process of step-by-step coevolution of metabolic pathways and amino acid repertoire of the proteins. These aspects of the coevolution theory of the origin of the genetic code are now more or less completely accepted. We now return to an early stage of this coevolution process, when a two amino acid repertoire evolved to a four amino acid repertoire, and see which implications this process had for an error-robust structure in the genetic code. 

**Binary Coding**. We see that at an early stage the protein coding system possibly was a binary system, with “alanine” *vs.* “glycine”: hydrophobic (“H”) *vs.* polar (“P”). Brack has found that alternating hydrophobic and polar residues form beta sheets, if salt is present [20]. In principle, (HHPP)*_n_* would mean “alpha-helix,” while (HPHP)*_n_* would mean “beta-sheet.” Of course, glycine is not the best example of a polar amino acid (because glycine gives so much freedom to move to the string of amino acids), and alanine has only restricted hydrophobic character. But this demonstrates the evolutionary opportunity available for amino acids like valine and aspartic acid. A thorough mathematical framework for a gradual, symmetry-breaking pathway to get from a restricted code to the present-day genetic code can be found in [21,22]. It is important to realize that as soon as tRNA genes had come into existence, a process of coevolution between the tRNA genes and the mRNA genes started. If, as an example, both the original anticodon for alanine and the one for glycine had C as the third nucleotide, natural selection would favor codons which started with G. If we assume that the middle positions of the anticodons are evolutionary the most stable, we can envision an early stage in life when most codons would be of the form GCN and GGN, a minority of the codons would be of the form HCN and HGN (where H stands for U or C or A), and codons of the form NUN and NAN would originally have been rare. Although rare, mutation caused them to appear in sequences, and if they did, they were read NUN as alanine and NAN as glycine. When new tRNAs emerged which could read NUN and NAN more effectively, the new assignments had to be “alanine-like” and “glycine-like” (because they were already present, at low frequency, in coding sequences), and valine and aspartic acid respectively fulfill these requirements.

**Diverse Dipeptides**. When further amino acids than alanine and glycine became part of the Set of Encoded Amino Acids, other catalytic dipeptides than GlyGly could become part of life’s coded dipeptide vocabulary. Gorlero *et al.* [23] drew attention to the fact that SerHis is doing a much better job at peptide bond formation than GlyGly. Shimizu reported chemical experiments in which ValAsp played a primitive synthetase role [24]. It is interesting that in the SIPF reaction AspVal emerges much more abundantly than ValAsp [8]. Thus, to take advantage of the properties of ValAsp discovered by Shimizu, life had to have been progressed from uncoded peptide synthesis towards coded dipeptide synthesis.

**Lipids and membranes**. A very different aspect of coded oligopeptide formation which could have been very important in the early RNA world was lipid formation. Lipids probably were essential in protecting biopolymers, keeping the gene products close to the genes and making the feedback loop possible, and also in enabling sophisticated enzyme-free RNA synthesis (see [6], and references therein), which was important when oligopeptides were only providing protection to RNA (by binding Mg^2+^ ions [15]). Zhang has found that oligopeptides containing several valine residues in the middle and aspartic acid at the C-terminus behave like lipids and organize themselves in membranes [25]. Given the difficulty which hydrophobic amino acids like valine and leucine show to function in the attacking role in the SIPF reaction [8,9,10], coded oligopeptide synthesis is the only way for the early RNA world to have these kind of lipids in large amounts. In a stage of life in which it has depleted prebiotically formed lipids and has not yet developed fatty acid synthesis, the molecules studied by Zhang could have sustained growth and could have been the major membrane components. Again the argument that the exact sequence is not that important (we encountered this point when we considered oligopeptides as storage compounds) is important here. Valine, leucine, threonine, proline, alanine—all these amino acids could have enough hydrophobic character to be able to function in the middle of these lipids. This is important in an age when precise coding is still something of the future.

In summary, we see that the relationship between proteins and nucleic acids (the first small genetic code) and the emergence of macromolecular sequences (ribozymes) are closely connected, in this view of the emergence of life, with the origin of biological catalysis and the emergence of biological building blocks. We see that just the emergence of large RNA macromolecules per se, without peptides and lipids present, is considered in this review to be not realistic, and that therefore peptides, lipids, and small RNAs should be seen as co-evolving instead of just evolving right from the start. Finally, we see that many special events have to happen at the same time and at the same location leading to the emergence of the first prokaryotic cell. One could say that the emergence of life, of that special feedback loop, is a miracle. However, that is not the scientific way to express the situation. The scientific way to express the situation is that the origin of life was a very improbable event.

## 4. Error Robustness

The general idea behind Section 3 is that the start of things had to be simple instead of complex. To first have long, complex proteins, and subsequently introduce a way of coding them, looks absurd from an evolutionary biological viewpoint. Biological evolution does not work in that manner. The appropriate chronology of events is to first develop a coding system, use this for the production of relatively simple things, and, subsequently, develop large polypeptides. The start had to be simple—that is the idea. An intermediate stage, between the coding of simple dipeptides and the coding of large proteins, is the stage in which coded peptide synthesis produced things like heptapeptides. We already touched upon a very important one, AsnAlaAspPheAspGlyAsp, the universal active site making mRNA. To encode this oligopeptide 21 nucleotides in RNA are needed, and of course also the rRNAs and dipeptides, necessary for the system to work, had to be present. This means that quite a lot of RNA sequence had to be reproduced, which brings us to the topic of error robustness.

Especially during the start of life, the error catastrophe [26] was a problem. To get faithful replication, in principle many components were necessary, but at the same time, unfaithful replication could replicate only a restricted amount of sequence. To have many different kinds of amino acids when peptides have not yet reached a stage much longer than seven residues may not have been possible. Iyer *et al.* [27] show a solution to this problem in their Figure 1. Although the mRNA producing heptapeptide sequence in RNA polymerase is universal, related sequences (able to do RNA polymerization) are known from bacteriophages and from eukaryotic gene silencing. An early eukaryote probably picked this protein up by lateral gene transfer from a virus [27], and ancient viruses have probably saved these sequences, stemming from the distant past, from extinction [27]. The residue of phenylalanine turns out to be quite variable in these sequences: sometimes the place is taken by methionine, sometimes by leucine, and a single time by tyrosine. All these residues are bulky, and relatively hydrophobic. One can envision how this position in the sequence, sticking out like a pan-handle away from the active site [12], originally was occupied by valine, or alanine. Asparagine is of course a specialized form of aspartic acid. In this way, the active site could originally have been AspAlaAspValAspGlyAsp, or AspAlaAspAlaAspGlyAsp. Having a system to encode three or four different kinds of amino acids therefore probably was sufficient to produce a proto-version of the universal heptapeptide. If different amino acids can function in the same position, as seen in the bulky residue position just discussed, this provides of course error robustness when the codons for the different amino acids are similar. The valine and alanine codons are a good example.

Important contributions to the problem of preventing error catastrophe were made during the last years with [28,29]. The key idea here is that mutant products connected to the template string with mismatches, reproduce far slower than products connected to the template with just correct Watson-Crick matches [28]. The mutants appear, but they do not really reproduce. They lose in the competition from faithfully reproduced sequences. Worse, mismatches lead to more mismatches, slowing their reproduction further, a circumstance known as an error cascade [29]. Because of these kinds of effects, developing life is able to get around the error catastrophe. These considerations apply emphatically to enzyme-free RNA replication, and are therefore particularly important during the era bridging the age in which aspartic acid containing oligopeptides were only having an RNA-protecting function and RNAs were very small with the age in which aspartic acid containing oligopeptides were starting to run RNA synthesis. As has been mentioned before, lipids also play a role in this chronology (see [6] and references therein).

The presence of rare, near-cognate codons in genomic sequences discussed above in Section 3, plays a role in the further building up of error robustness in the genetic code. Higgs [30] pointed out the four-column structure of the genetic code. When GUN codons originally encoded valine but HUN codons were used for coding valine every now and then, a perfect starting point was present to, for example, enlarge the coding system with leucine coded by CUN. In this way, middle-U codons became hydrophobic amino acid encoding, middle-C codons became small amino acid encoding, and middle-A codons became polar amino acid encoding. In [14] it was pointed out that the fourth column also has a special characteristic: nucleic acid binding. This is what both glycine and arginine do. The U-starting codons seem to be less bound to these column characters than the other 48 codons. With help of the error robustness, functional products could be made in an age during which the error catastrophe prevented the existence of very large genome memory space, necessary for encoding the protein products which are required for very precise production of proteins consisting of 20 amino acids. An early publication pointing out this essential aspect of the origin of life is [31]. Probably, the U-starting codons acquired their assignments when the system had already progressed from the stage of impossibility of precise replication.

## 5. The Information Frozen in the Genetic Code

Having pointed out (1) the improbability of finding extraterrestrial life, (2) the improbability of the emergence of life, and (3) the difficulty of getting around the error catastrophe, it is clear that the only source which can give us information on the emergence of life (apart from laboratory experiments, see e.g. [6] and references therein, and computer simulations) is present-day life on Earth. In the biological constitution of organisms, information concerning their origin is sometimes frozen. A well-known example of such frozen information is the fact that whales have a rudimentary pelvis and rudimentary hind leg bones. This gives us information that once the ancestors of whales were four-legged animals. In the same way as anatomy can provide us with such information, so can biochemistry. As an example, the basic structure of the standard genetic code is a biochemical feature which contains such information. Eight of the codon boxes (groups of four codons of which the first and the second nucleotide are shared) are fourfold degenerate (please consult your genetic code table). Of the remaining eight codon boxes, all Y-ending pairs are twofold degenerate (please consult your genetic code table again). In not a single case is an A-ending codon the unique codon for an amino acid (please consult your genetic code table once more). Unmodified C in the first anticodon position does not wobble [32]. Unmodified G in the first anticodon position recognizes both Y-ending codons [32]. Unmodified U in the first anticodon position recognizes all four codons rather efficiently, but only in the eight fourfold degenerate codon boxes [33]. These six facts together give us the information that at a certain stage in the development of life (well before the last universal common ancestor) a tRNA set containing 8 U-starting anticodons, 8 G-starting anticodons, and 8 C-starting anticodons existed [14,34] which produced remarkably complete (in terms of number of amino acids) and unambiguous genetic coding. It is this kind of biochemical facts which gives us information concerning the origin of life, not space exploration.

## 6. Concluding Remarks

This study is not the first to point out that enthusiasm about extraterrestrial life is misplaced when all that is found is a simple compound. Earlier this year, Narita *et al.* [35] made the same point in connection to molecular oxygen. Fluid water is very special, but our beautiful Earth-Moon-Double-Planet-System has more aspects that are special. The Earth-Moon-Double-Planet-System is a rare oasis in a barren Universe. Something to handle with care—we have one only.

Since Pasteur, we no longer believe that mice or bacteria can very easily develop from just dead dirt. The present review has tried to point out what it is exactly which makes living organisms so special. It has been argued that from a very early stage in prebiotic/biotic evolution, a feedback loop has been in existence in which peptides and RNA were both involved. The circumstances which created that feedback loop (lipid presence among them) might have been very rare on the scale of the Universe.
